# A Depth-Based Fall Detection System Using a Kinect^®^ Sensor

**DOI:** 10.3390/s140202756

**Published:** 2014-02-11

**Authors:** Samuele Gasparrini, Enea Cippitelli, Susanna Spinsante, Ennio Gambi

**Affiliations:** Dipartimento di Ingegneria dell'Informazione, Università Politecnica delle Marche, Via Brecce Bianche 12, Ancona 60131, Italy; E-Mails: e.cippitelli@univpm.it (E.C.); s.spinsante@univpm.it (S.S.); e.gambi@univpm.it (E.G.)

**Keywords:** depth frame, elderly care, fall detection, human recognition, Kinect

## Abstract

We propose an automatic, privacy-preserving, fall detection method for indoor environments, based on the usage of the Microsoft Kinect^®^ depth sensor, in an “on-ceiling” configuration, and on the analysis of depth frames. All the elements captured in the depth scene are recognized by means of an *Ad-Hoc* segmentation algorithm, which analyzes the raw depth data directly provided by the sensor. The system extracts the elements, and implements a solution to classify all the blobs in the scene. Anthropometric relationships and features are exploited to recognize one or more human subjects among the blobs. Once a person is detected, he is followed by a tracking algorithm between different frames. The use of a reference depth frame, containing the set-up of the scene, allows one to extract a human subject, even when he/she is interacting with other objects, such as chairs or desks. In addition, the problem of blob fusion is taken into account and efficiently solved through an inter-frame processing algorithm. A fall is detected if the depth blob associated to a person is near to the floor. Experimental tests show the effectiveness of the proposed solution, even in complex scenarios.

## 1. Introduction

One of the most important research activities related to the Ambient Assisted Living scenario is in the field of automatic fall detection. It is well known that in the case of a fall event, enabling fast help intervention allows decreasing the probability of complications derived from physical damage due to the accident (such as those caused by fractures of the lower or upper limbs) [1]. In the last decades, several automatic fall detection techniques have been proposed. In [2], wearable sensors are used to provide information about critical movements. The main drawback of the approaches based on wearable sensors, from the user's perspective, is the need to wear and carry various uncomfortable devices, during normal daily life activities. Non invasive (*i.e.*, not wearable) solutions are the subsequent step in resolving the problem of automatic fall detection. In [3], the authors used an RGB camera to monitor the environment, but in recent years depth cameras have become a new tool to exploit. The availability of depth information allows one to implement simpler identification procedures to detect human subjects. The advantages of this technology, with respect to classical video-based ones, are:

- less susceptibility to variations in light intensity;
- 3D information by a single camera, while a stereoscopic system is necessary in the RGB domain to achieve the same goal;
- privacy issues: it is not possible to recognize the facial details of the people captured by the depth camera. This feature helps to keep identity confidential.

Several commercial devices are able to provide depth data at a stable frame rate. One of these is the Time Of Flight (TOF) camera, as used in [4], that can retrieve the distance information by evaluating the photons' propagation time along the distance between the sensor and the intercepted objects.

The Kinect^®^ for Windows sensor [5], used in this work, exploits the “structured light” approach. The depth information is generated by analyzing the distortion of an infrared pattern, projected by the sensor and scattered by the surface of the intercepted objects. The sensor was designed for gaming and recreational purposes, with the aim of intercepting body movements, when placed at a height of 60 ÷180 cm from the ground. However, the scenario in which we want to use the device features an “on-ceiling” configuration of the sensor, meaning that it is facing downwards, to provide a complete top view of the scene. This specific configuration does not allow it to activate known skeleton tracking tools, like the NITE middleware [6], or Microsoft Skeleton [7], provided by OpenNI libraries and Microsoft SDK, respectively, that are usually exploited in classical, front-view situations. It is then necessary to develop an *ad-hoc* algorithm for automatic fall detection, based on the analysis of raw depth data provided by the sensor.

The logical steps upon which the proposed solution is built are:

- *preprocessing and segmentation*: the incoming depth frame is pre-processed to enable the subsequent steps. In this phase, a reference frame is generated to help better identification of human subjects;
- *distinguish object algorithm*: the algorithm identifies, splits, and classifies the different clusters of pixels in the frame;
- *identification of human subject*: starting from the set of separated objects, the system recognizes those representing a human subject, by evaluating several anthropometric relations;
- *people tracking and fall detection*: the system tracks the movements of the human subjects in the depth frames, and detects if a fall occurs. In addition, it is able to manage blob fusion when two or more subjects get in contact.

**Figure 1.**
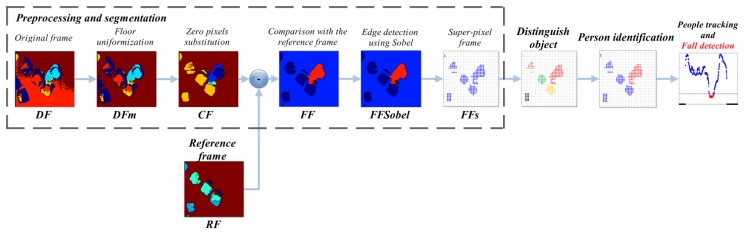
Overview of the fall detection system.

The entire procedure is illustrated in Figure 1, where each step described above can be identified. It might also be noted that the *preprocessing and segmentation* part is detailed with the outputs of every sub-step. The paper is organized as follows: Section 2 provides an overview of related work about automatic fall detection using depth frames. Section 3 describes the implementation details of the proposed system, while Section 4 underlines the performances achieved by the proposed approach. Finally, Section 5 summarizes the conclusions of the work.

## 2. Related Works

Many feature representation methods have been developed, based on color cameras, to recognize activities and actions from video sequences. The advent of the Kinect^®^ has made it feasible to exploit the combination of video and depth sensors, and new tools, such as the human activity recognition benchmark database [8], have been provided, to support the research on multi-modality sensor combination for human activity recognition. This paper focuses on the use of the depth information only, to realize automatic fall detection at the lowest complexity, for which different approaches have been proposed in the literature.

In [9], the Kinect^®^ sensor is placed on the floor, near a corner of the bedroom. A restriction of this setup is the limited coverage area, caused by the presence of the bed. A specific algorithm is proposed to handle partial occlusions between objects and the person to monitor.

Complete occlusions, due to the presence of bulky items (suitcase, bag, and so on), are considered within the paper, but they represent very common situations in true life. Another setup is described in [10], where the sensor is placed in standard configuration (60÷180 cm height from the floor), as recommended by Microsoft. The NITE 2 software is exploited to generate a bounding box which contains the human shape. The geometrical dimensions of this box are monitored frame by frame, to retrieve the subject's posture, and to detect falls. This solution is robust to false positive errors, *i.e.*, the generation of an alarm signal associated to a fall event is avoided, when the subject slowly bends over the floor, or picks up an object from the ground. The algorithm only deals with tracking the subject, whereas his identification is left to the NITE 2 software. Consequently, the NITE 2 skeleton engine constrains the system to support the minimum hardware specifications required by the SDK.

The authors in [11] present a different configuration, where the Kinect^®^ sensor is placed in one of the room top corners, and it is slightly tilted downward. Comparing the latter solution to the previous one, the coverage area obtainable is larger, but further data processing is necessary, to artificially change the point of view from which the frame is captured. This operation consists in a translation and a rotation applied on the system coordinates, to obtain a virtual top view (defined “height map”), very similar to the original frame provided directly by our approach, where the sensor is located on the ceiling. The computation of the transform parameters needs a preliminary manual identification of some reference points on the floor. In [12], the top view is obtained using a self-improving method, whereas in [13] the authors derive another solution based on a so-called “V-disparity image” technique. Similarly to the configuration previously discussed, the problem of partial occlusions can still exist. One of these situations occurs, for example, when the subject to monitor is behind a bulky object, like a couch or an armchair.

Taking into consideration the techniques described above, our solution has the following advantages:

- the top view depth frames are directly available, without the need of a transformation process applied to the spatial coordinates;
- the direct top view allows a better monitoring of the scene, than the ones in [9,13], and the occlusion phenomenon is therefore reduced;
- avoiding a machine learning solution in our approach strongly reduces the computational demand;
- the algorithm is portable on different hardware platforms, as it works on raw depth data, possibly captured by different sensors, not only Kinect^®^. This is not the case for the system proposed in [10], which is bound to the NITE 2 middleware.

## 3. The Proposed Method

The system setup adopts a Kinect^®^ sensor in top view configuration, at a distance of 3 m (*MaxHeight*) from the floor, thus providing a coverage area of 8.25 m^2^. To extend the monitored area, the sensor can be elevated up to around 7 m; beyond this distance the depth data become unreliable. The algorithm works with raw depth data (given in millimeters), that are captured at a frame rate of 30 fps with a resolution of 320 × 240 pixels, using Microsoft SDK v.1.5.

### 3.1. Preprocessing and Segmentation

**Figure 2.**
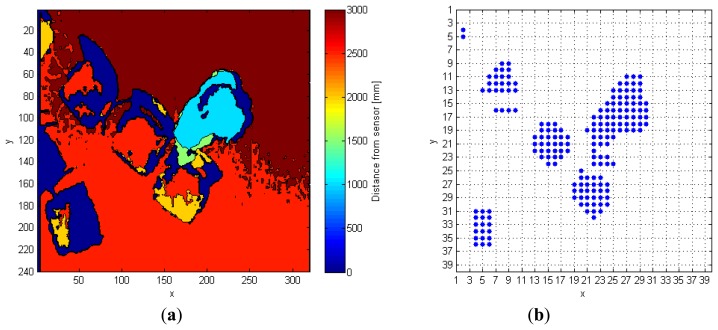
(**a**) Depth frame (*DF*) input to the system, and (**b**) foreground frame (*FF*) input to distinguish object algorithm.

The input depth frame (*DF*) is represented in Figure 2a. As discussed in Section 2, the operation of floor identification implemented in our system is simpler than the solutions proposed in [11–13]. In *DF*, all the pixels for which the difference of their depth value from the *MaxHeight* value is within the range of 200 mm, are set as belonging to the floor surface, thus obtaining a modified depth frame (*DFm*). This range is empirically evaluated, and it depends on the *MaxHeight* value. When the sensor cannot evaluate the depth information for some pixels, as those corresponding to corners, shadowed areas, and dark objects, it assigns them a null depth value. There are various approaches to handle null depth values: differently from [9], where the null values are discarded, in [14] the authors propose a substitution process. In [15], a so-called “flood-fill” algorithm is used to resolve this problem, while in this work the null pixels are replaced by the first valid depth value occurring in the same row of the frame.

The frame called *Current Frame* (*CF*) is the output of this substitution operation. At this point, a comparison between *CF* and a reference frame (*RF*) generates a foreground frame (*FF*), that emphasizes the pixels belonging to the human shape. The *RF* is very similar to the *CF*, but it contains only still objects, without any human subject, as it is captured in the initial phase, when the sensor starts catching depth frames, and no people must be in the scene. Equation (1) defines the value of the pixels in the FF:

(1) $$FF\left(x,y\right)=\{\begin{array}{cc} CF\left(x,y\right)+\text{gapCoeff} & if\left|CF\left(x,y\right)-RF\left(x,y\right)\right|>\text{ThPerson}\\ CF\left(x,y\right) & \text{otherwise} \end{array}$$

where *x* is the column index and *y* is the row index of the pixel in the frame; the *ThPerson* threshold is set to 50 mm, and it allows identification of depth gaps that reveal new objects, or human subjects, in the scene. The pixels that verify the first condition in Equation (1) are increased by the *gapCoeff* quantity: this addition can be defined as a *Depth level slicing*, similar to the *Intensity level slicing* process in [16]. The latter method is used to enhance the relative visual perception on RGB images, while, in this context, it enables to improve the object discrimination step. A *Sobel edge detection* solution helps to achieve objects separation inside the scene, especially when they are overlapped. The object bounds extracted are then set to the floor depth level (*MaxHeight*), in the *FFSobel* frame, according to Equation (2):

(2) $$\text{FFSobel}\left(x,y\right)=\{\begin{array}{cc} \text{MaxHeigth} & if(\text{Sobel}(CF\left(x,y\right))<\text{ThSobel})\\ FF\left(x,y\right) & \text{otherwise} \end{array}$$

The Sobel algorithm computes an intensity gradient for each pixel in the *CF*. The output value must be compared to a threshold, to set the level of detail of the edges. This threshold, named *ThSobel*, is empirically set to 2,000. Based on both Equations (1) and (2), setting the parameter *gapCoeff* equal to 6,000 mm allows maintaining *ThSobel* fixed, and ensures the correct discrimination of the human shape, even when it features depth values very similar to those of nearby objects.

The last operation consists in the creation of a so-called 40 × 40 super-pixel frame (*FFs*): each super-pixel corresponds to a 6 × 8 block of pixels in *FFSobel*. The *i*-th super-pixel takes the value 1, if all the pixels in the block differ from *MaxHeight*, otherwise it takes the value 0. This process improves the separation between each object in the scene, and also allows the processing time to decrease because the total amount of pixels passed to the following steps is reduced, as shown in Figure 2b.

### 3.2. Distinguish Object Procedure

This section describes the discrimination algorithm that splits all the objects present in the depth scene. The frame resolution value required by the procedure is not fixed, so the algorithm can work with different depth frame sources. The sorted coordinates of each object in the area represent the output of the distinguish object procedure. The data structure containing this information is called *Mat_Obj*, and shows, for each row, the coordinates of all the super-pixels associated with a single object. The idea behind the algorithm is to scan one row at a time, to discern the coordinates of single *object parts* (*OP*) corresponding to the same blob. Two *OPs* in the same line are classified as belonging to separated objects, if there is at least a null value between them. The column indexes of each *OP* are saved in the *Mat_RowObj* structure, where each row is related to a different blob, and contains the following information:

- first column index of *OP*;
- last column index of *OP*;
- object identifier (OI) within the *Mat_Obj* structure (0 value denotes that the *OP* is not yet classified).

The *Mat_RowObjOld* variable has the same structure described above, but it refers to the row analyzed in the previous step. The algorithm compares each *OP* inside *Mat_RowObj* and *Mat_RowObjOld*, to correctly populate the *Mat_Obj* structure. The comparison between *OPs* in different rows is performed by means of two row vectors, called *CompareVector* (*CV*) and *CompareVectorAllOld* (*CVo*), of the same width of the input matrix. In these vectors, values 1 represent *OP*, whereas values 0 denote the floor. Each group of *OP* super-pixels identified in the current row is loaded individually within *CV*, by exploiting the information found in *Mat_RowObj*. *CVo* instead, stores an exact copy of the row previously analyzed. The next step is a *bitwise and* operation between *CV* and *CVo*; a not-null result means the continuation of the object in the current line, otherwise a new object has been found.

The following example shows a practical application of this approach. The input frame, *FF* in Figure 2b, contains seven different objects that must be classified separately. In details, after the analysis of the fifth line, the variables *Mat_RowObj* and *Mat_RowObjOld* are populated as follows:

(3) $$\begin{array}{cc} \text{Mat}\_\text{RowObj} & =\left[\begin{array}{ccc} 2 & 2 & 0 \end{array}\right] \end{array}$$

(4) $$\text{Mat}\_\text{RowObjOld}=\left[\begin{array}{ccc} 2 & 2 & 1 \end{array}\right]$$

The overlap of column indexes in the above structures leads to adding the element *OP* [2:2] in the first row of *Mat_Obj* (as imposed by the value in the third column of *Mat_RowObjOld*). Similarly, at the thirteenth row:

(5) $$\begin{array}{cc} \text{Mat}\_\text{RowObj} & =\left[\begin{array}{lll} 5 & 10 & 0\\ 25 & 29 & 0 \end{array}\right] \end{array}$$

(6) $$\text{Mat}\_\text{RowObjOld}=\left[\begin{array}{lll} 6 & 10 & 2\\ 26 & 29 & 3 \end{array}\right]$$

In this case, *OP* [5:10] is classified as a part of object 2, while [6:10] as object 3.

Table 1 shows the output results of the algorithm; the seven objects located in the input *FF* frame have been correctly separated from each other. Due to the large number of super-pixels present inside some objects, only the first five *(x,y)* couples of coordinates are displayed.

**Figure 3.**
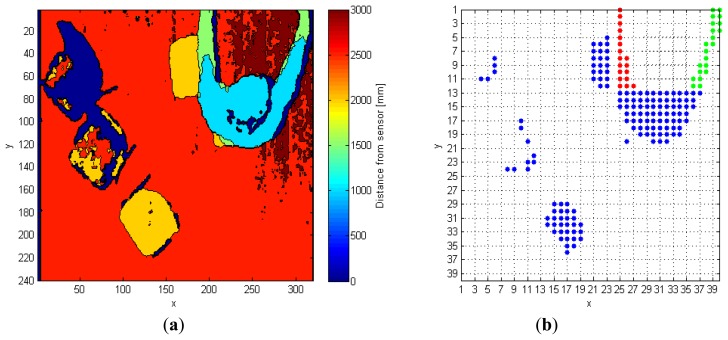
Possible side-effect condition: (**a**) in the DF, and (**b**) in the FF frame, input to distinguish object algorithm. In (b), arms are highlighted with different colors.

The algorithm must account for possible side effects, as the one depicted in Figure 3a, that shows a person with outstretched arms. The outstretched arms, taken as objects in the depth frame, are represented by different colors until row 13 (Figure 3b), because it is not possible, until this row is processed, to associate them to the same object. The algorithm can identify this condition, because the indexes of many *OPs* in *CVo* match the same *OP* in *CV*. In details, looking again at the thirteenth row in Figure 3b, *bitwise and* operation between *CV* and *CVo* gives 1 in columns 25-26-27 (left arm), and in columns 36–37 (right arm). As a consequence, it is necessary to associate the three *OPs* (arms and body) to the same object. This correction also applies when the collapsing *OPs* are more than two. The entire procedure discussed in this subsection is summarized in Algorithm 1.


**Algorithm 1.** Distinguish object procedure.
**Input**: *FFs* frame**Output**: *Mat_Obj* matrix1 **for** each row in Input frame **do**2 Construction of *Mat_RowObj* structure3  **for** each element in *Mat_RowObj*4   **if** current row index > 15    Construction of *CompareVector*6    *BitWise_Res* = *CompareVector* & *CompareVectorAllOld*7    **if**
*BitWise_Res* is not null8     connect the actual *OP* to the corresponding previous object9    **else**10     classify the actual *OP* as a new object11    **end if**12   **else**13    classify the actual *OP* as a new object14   **end if**15   **if** side-effect16    *Mat_Obj* reordering17   **end if**18  **end for**19 *Mat_RowObjOld* = *Mat_RowObj*20 *CompareVectorAllOld* = all *OP* in actual row21**end for**


### 3.3. Identification of Human Subjects

For each object, the corresponding central point is evaluated, by averaging row and column indexes. The *i*-th *central super-pixel* (s*cp_i_*) is taken as the super-pixel of the *i*-th object closest to the midpoint computed; this restriction improves the tracking process of the human subject in subsequent frames, because it keeps the s*cp_i_* within the subject. The coordinates of s*cp_i_* are mapped to the 320 × 240 pixel frame, and they take the name of *cp_i_*. In addition to the midpoint *cp_i_*, the system identifies the maximum height value in the *i*-th element. In order to localize the point featuring the maximum height value, that should reasonably result close to the center of the subject's head, the algorithm performs an average operation on the pixels located inside specific distance and depth ranges from the maximum. The resulting point of this operation is indicated with *mp_i_*.

The function *IsPerson* takes as an input the *mp_i_* coordinates information, and recognizes if that blob belongs to a person or not. The research method, implemented by *IsPerson*, exploits the anthropometric relations that characterize the human body [17]. For each object, the features evaluated starting from the *mp_i_* point are:

- head-ground distance gap;
- head-shoulder distance gap;
- appropriate head dimension.

**Figure 4.**
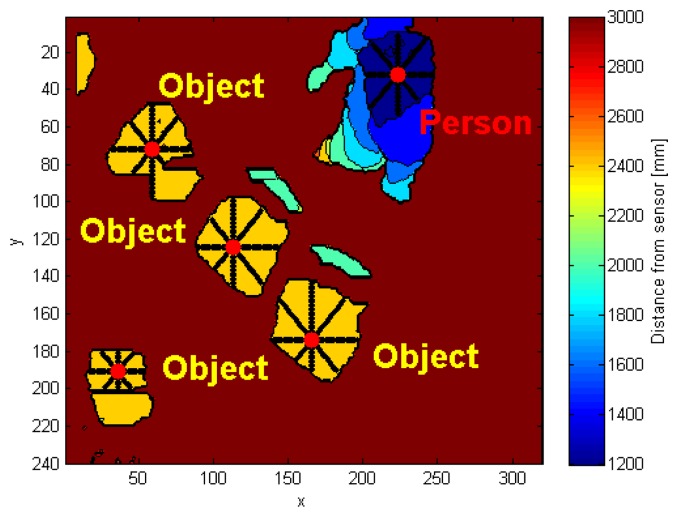
Research on the human subject in *CF*.

The function considers the depth information, starting from *mp_i_* (dots within each object area in Figure 4, and moving towards the eight cardinal directions, by steps of single pixel. The depth difference values between two adjacent points are evaluated, for each direction. These values are called *DiffCons*, and used to evaluate the selected features.

#### 3.3.1. Head-Ground Distance Gap

The top-view setup of the sensor makes it possible to evaluate the depth difference that occurs when passing from a head pixel, to another that belongs to the ground surface. This gap could be not verified by some generic objects. Consequently, the control excludes from the group of possible human subjects the blobs that do not verify the following condition:

(7) $$\begin{array}{ll} if\left(\text{DiffCons}\geq \frac{CF(r_{i},c_{i})}{2}\right) & \left(r_{i},c_{i}\in mp_{i}\right)\\ \;\text{head}-\text{ground gap found}\\ \;\text{else}\\ \;\text{head}-\text{ground gap not found} \end{array}$$

#### 3.3.2. Head-Shoulder Distance Gap

Another feature of the human shape evaluated by the proposed algorithm is the depth difference that can be found between head and shoulders. Based on the test campaign carried out by the authors in [17], height possible values of the head dimensions are included in the 20–30 cm range. This range is the result of the difference between the human height (number 805 in [17]), and the shoulders height (number 841). Taking into account the human silhouette, the algorithm requires this condition to be verified at least in two directions, out of the total eight ones.

#### 3.3.3. Appropriate Head Dimension

The algorithm computes the pixels distance between the *mp_i_* point and the coordinates that verify conditions expressed in Sections 3.3.1 and 3.3.2. The directions that do not meet the depth gap requirements take on a value equal to the distance from *mp_i_* to the frame boundary, in that specific direction. Figure 4 shows, starting from the *mp_i_* point in each object, the eight vectors related to the directions: N, NE, E, SE, S, SW, W, NW. Each distance value, associated to one of the four principal directions (N, NE, E, SE), is added to its opposite, respectively S, SW, W, NW. The four resulting quantities correspond to the pixel segments shown in Figure 4, which cross *mp_i_*. They are converted into real distances, by using the equations in [18], in order to obtain a spatial information independent from depth measurement.

**Figure 5.**
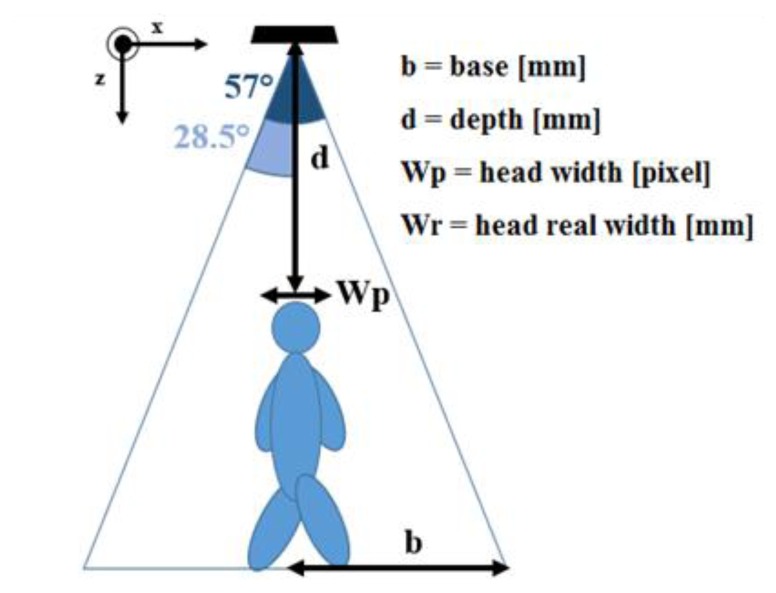
Side view of the system configuration.

Figure 5 shows the computation of the real distance (*Wr*) corresponding to a pixels segment (*Wp*), which is parallel to the *x* axis. The theory of right-angled triangles is used to derive the base length, defined as *b* parameter in Figure 5, from the depth information provided by the Kinect sensor *d* (mm), and its angle of view. The *Wr* variable is obtained by a proportion based on the total number of frame pixels along the *x* direction (320), *Wp* and *b*:

(8) $$b=d\cdot \tan(28.5{}^{\circ}),\frac{Wp}{320}=\frac{Wr}{2\cdot b}\Rightarrow Wr=\frac{2\cdot b\cdot Wp}{320}$$

Equivalently, if the segment belongs to the *y* direction, it is necessary to replace 320 with 240 pixels, and 28.5° with 21.5° for the angle of view, according to the sensor specifications. Finally, when the segment is not parallel to *x*-axis nor *y*-axis, it is necessary to split the computation into its orthogonal components, according to:

(9) $$Wr=\sqrt{{\left(\frac{2\cdot d\cdot \tan(21.5)\cdot Wp}{240}\right)}^{2}+{\left(\frac{2\cdot d\cdot \tan(28.5)\cdot Wp}{320}\right)}^{2}}$$

The computation of real distance is applied to all the four directions. The resulting quantities must respect the reference values in [17], in order to recognize a person's head. In particular, the head diagonal dimensions must be comparable each other, and they must belong to the 20–40 cm range. This range was also derived from different measurement campaigns, published as numbers (441) and (702) in [17].

The data structure *CoordPerson* stores the most important information of the objects that pass the previous controls, and potentially correspond to human subjects. This structure has a different entry for each subject, and contains:

- *cp_Per_* (*x*-*y*) pixel coordinates of *cp_i_*;
- s*cp_Per_* (*x*-*y*) super-pixel coordinates of *scp_i_*;
- *mp_Per_* (*x*-*y*) pixel coordinates of *mp_i_*;
- *smp_Per_* (*x*-*y*) super-pixel equivalent coordinates of *mp_i_*.

A similar structure, called *CoordObj*, is used to store the information of blobs classified as objects:

- *cp_Obj_*: (*x*-*y*) pixel coordinates of *cp_i_*;
- s*cp_Obj_*: (*x*-*y*) super-pixel coordinates of *scp_i_*;
- *mp_Obj_*: (*x*-*y*) pixel coordinates of *mp_i_*;
- *smp_Obj_*: (*x*-*y*) super-pixel equivalent coordinates of *mp_i_*.

### 3.4. People Tracking

During the frame processing operations, the function *IsPerson*, described in Section 3.3, is called only on the blobs that have not yet been classified as human subjects in preceding frames. On the other hand, the blobs already recognized as human subjects are tracked, in order to identify if a fall occurs.

The computationally simplest solution to perform human tracking is based on the evaluation of the Euclidean distance between *scp_Per_*, obtained from the (*k−1*)-th *FF* frame, and the *scp_i_* of each blob within the *k*-th frame. However, experimental tests showed that this approach can lead to wrong results, especially when the frame rate drops, or several subjects, somehow in contact with each other, are merged in the same frame as a unique blob. There is the need to consider a new smart solution to manage possible complications.

**Figure 6.**
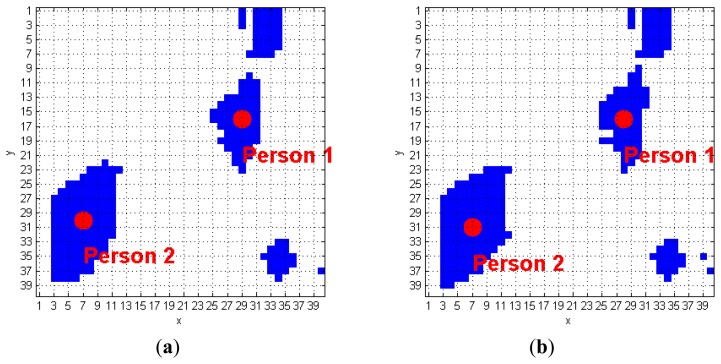
Consecutive *FF* frames, with recognized human targets: (**a**) *(k−1)*-th frame, (**b**) *k*-th frame.

**Figure 7.**
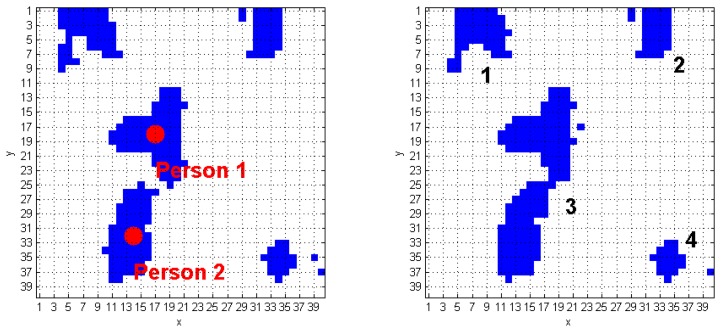
Blob fusion between two human subjects: (**a**) *(k−1)*-th *FF* frame, (**b**) *k*-th *FF* frame.

The algorithm we developed to track human subjects from the captured depth frames starts by placing the person position (*scp_Per_*), computed over the (*k−1*)-th *FF* frame, inside the *k*-th *FF* frame. The blob that contains this point is automatically associated to a human target. Figure 6 shows a simple application of this approach: it the *(k−1)*-th *FF* frame, the *scp_Per_* point of the human target labeled as 1 is positioned in (29, 16), and, at the *k*-th *FFs*-frame, that point is again inside the same blob. The next step requires updating the *scp_Per_* coordinates, according with the procedure described at the beginning of Section 3.3. The result, again for blob 1 of the previous example, is (28, 16), as shown in Figure 6. The same operation is applied on the blob labeled as 2, thus passing from the coordinates (7, 30) to (7, 31). When more human subjects are close together, they may be included in the same blob. These situations may occur due to partial superimposition of silhouettes, as in a shoulders contact. The fusion effect occurs because the people's shoulders may take very similar height values, and the Sobel algorithm fails. Figure 7 evidences this phenomenon; in detail, the two human subjects correctly recognized in the (*k−1*)-th *FF* frame, get merged into the same blob, labeled as 3, in the *k*-th *FF* frame.

This situation can be fixed by an *ad-hoc* procedure, able to detect fusions, and to properly separate people and objects. The first step consists in filling in a data structure of size *B*x4, called *TrackingInfo*, exploiting the information stored in *CoordPerson* and *CoordObj*. *B* denotes the number of blobs identified in *k*-th *FF* frame. The *b*-th entry of the *TrackingInfo* structure contains the following fields:

- *Pers*: identifiers (assigned at (*k−1*)-th *FF* frame) of the human subjects found in the *b*-th blob;
- *Obj*: identifiers (assigned at (*k−1*)-th *FF* frame) of the objects found in the *b*-th blob;
- *N_Pers*: number of identifiers in *Pers* field;
- *N_Obj*: number of identifiers in *Obj* field.

This classification allows checking one of the following conditions:

- *N_Pers* equals 0 and *N_Obj* is less than, or equal to 1 (*N_Pers* = = 0 && *N_Obj ≤* 1):
  This situation occurs when there are no human subjects that were already recognized in the (*k−1*)-th *FF* frame (*N_Pers* = 0), and a new object appears (*N_Obj* = 0). *N_Obj* = 1, instead, means that the blob represents an “old” object. In this case, there is no fusion, because the blob is composed by a single element, *i.e.*, an object. The function *IsPerson* is called to check if the object is actually a human subject that has not been yet recognized;
- *N_Pers* equals1 and *N_Obj* equals 0 (*N_Pers* == 1 && *N_Obj* == 0):
  A human subject, and no old object, is identified within the blob. Even in this case, fusion does not occur. Consequently, all the associated elements in *CoordPerson* are updated with the coordinates computed by taking into account the new shape of the blob, as visible in Figure 6;
- Otherwise
  A fusion occurred if the conditions *A* or *B*. are not met. This situation can refer to several human subjects or objects into the same blob *b*. Each element in the *b*-th blob must be separated and classified.

As visible in Figure 7a, there are four blobs inside the *k-th FF* frame, and the corresponding *TrackingInfo* table has four rows, as shown in Table 2.

The first row refers to the blob in the top-left corner, that matches condition A. It means that the first blob was already detected in the preceding frame. The same holds for the 2nd and the 4th rows of Table 2. Each of the previously discussed cases requires calling the function *IsPerson* that separately checks the presence of human subjects in the blobs. The blob in the 3rd row of *TrackingInfo* meets condition *C*, and this fact denotes that a fusion between two human subjects occurred.

**Figure 8.**
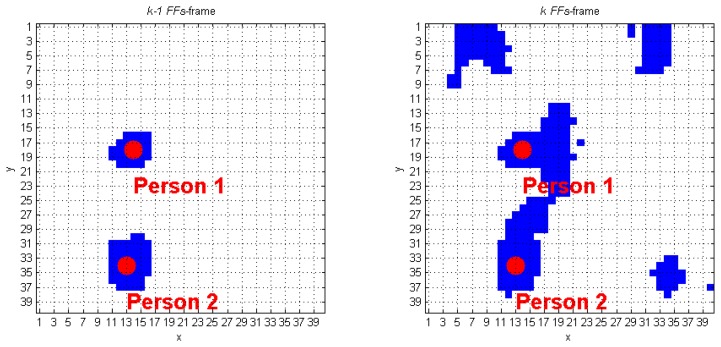
Handling the fusion of two human subjects into a single blob.

The algorithm discards all the super-pixels located outside a spatial range that starts from the *smp_i_* coordinates. This process is performed for each element that originates the fusion. The new blobs obtained from this process must be recognized and classified using the *DistinguishObject* function. This approach allows managing the fusion event shown in Figure 7b, and gives the results provided in Figure 8. The processing of the *k*-th frame ends by updating the *CoordPerson* and *CoordObj* data structures, that will be used to process the following frame.

Obviously, the procedure discussed above is able to manage not only the case of two subjects merging, but it can also work when several subjects interact with each other.

Once a human subject is tracked, the height information of *cp_Per_* in the *CoordPerson* data structure is evaluated, and the system detects a fall when this quantity gets similar to the value of the floor distance. The threshold value used to discriminate a fall event is set to 400 mm, which is experimentally evaluated as the thickness of the human body revealed by the sensor, when a person lies on the floor.

## 4. Performance Analysis

This section discusses the performances of the proposed technique for fall detection, based on processing the depth information provided by the Kinect^®^ sensor in top-view configuration. As a first evaluation of the fall detection system, we consider the simplest case: a single person walks in the scene, and falls to the ground, without interacting with objects (*i.e.*, the person does not intercept any object when walking or falling down).

**Figure 9.**
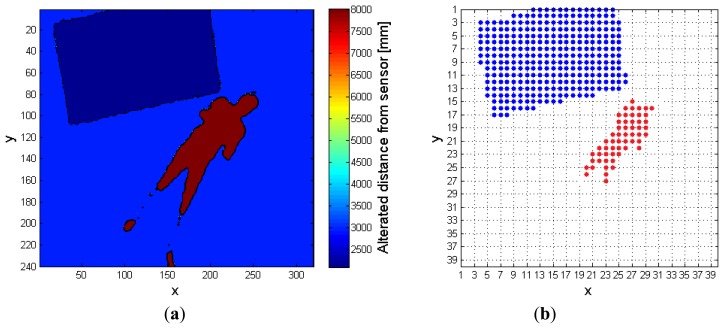
Simple fall, without objects interaction: (**a**) *FFsobel* frame, where human's pixels are enhanced, and (**b**) corresponding *FF* frame, where the red super-pixels refer to the person.

**Figure 10.**
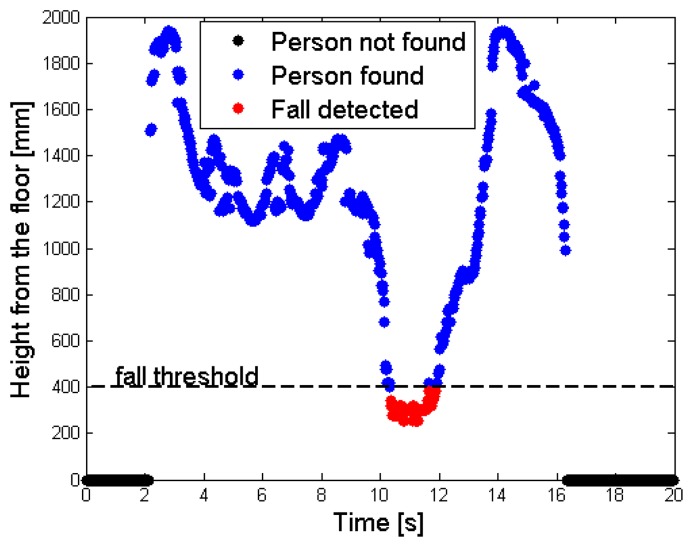
Time sequence of the depth values assumed by the person's central point (*cp_Per_* parameter), with fall event detected and highlighted in red.

Figure 9a shows this situation: a single desk is present in the scene, and the person has just fallen. The floor has a depth value of *MaxHeight* (light blue), whereas the unique object in the scene (the desk) features a smaller depth value, because it is nearer to the sensor. As explained in Section 3.1, the person does not belong to the *RF*, so the corresponding depth values are increased by the *gapCoeff* quantity. The legs in the human body are partially discarded in this setup, because the difference of their depth value from the *MaxHeight* does not exceed the range of 200 mm. Figure 9b highlights by means of red points the super-pixels associated to the person, in the *FF*. Finally, Figure 10 shows the different height values taken by the *cp_Per_* parameter, and stored inside the *CoordPerson* data structure along the time. The person is detected after about 2 s from the beginning of the test, due to the fact that initially he was outside the coverage area of the sensor.

The tracking phase starts as soon as the person is recognized (blue spots); the central point moves from the shoulders to the head, while the subject is approaching the center of the scene. This movement is visible in the time interval [2,3] s, as an increasing height from the floor value. After this time interval, the fall event takes place, and the height values in the graph drop down. When the height value detected undergoes the 400 mm threshold, the system triggers an alarm and the fall event is notified (red spots). In the test, the subject lies on the floor for about 2 s, then gets up and moves away from the area monitored by the sensor. The decreasing of the height from the floor value, that occurs before the person exits the area monitored by the sensor at time instant 16 s, is also due to the shift of the central point position inside the human subject's shape.

**Figure 11.**
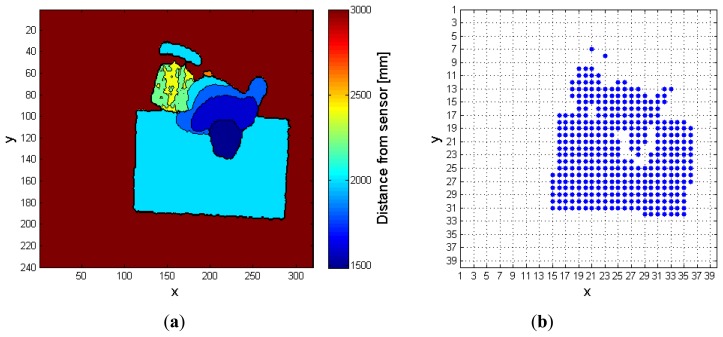
Complex fall with object interaction: (**a**) preprocessed frame, (**b**) corresponding *FF* frame. When the algorithm avoids using the *RF* and works on the *CF* only, the person is merged with the desk.

The second example, depicted in Figure 11, describes a more complex situation, in which the person to monitor interacts with a desk and a chair, before falling down. Differently from the previous case, it can be shown that, in this complex condition, the use of the *RF* is essential to reveal the fall. Figure 11a,b represents, respectively, the preprocessed depth frame, and the corresponding *FF* frame, when the *RF* is not exploited to extract the person. In the latter, the super-pixels associated to the human subject are wrongly merged with those belonging to the chair and desk blobs.

**Figure 12.**
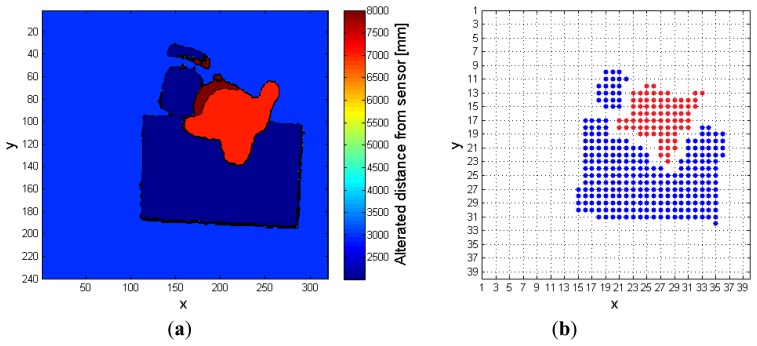
Complex fall with object interaction: (**a**) preprocessed frame, (**b**) corresponding FF frame, when the algorithm uses the RF.

**Figure 13.**
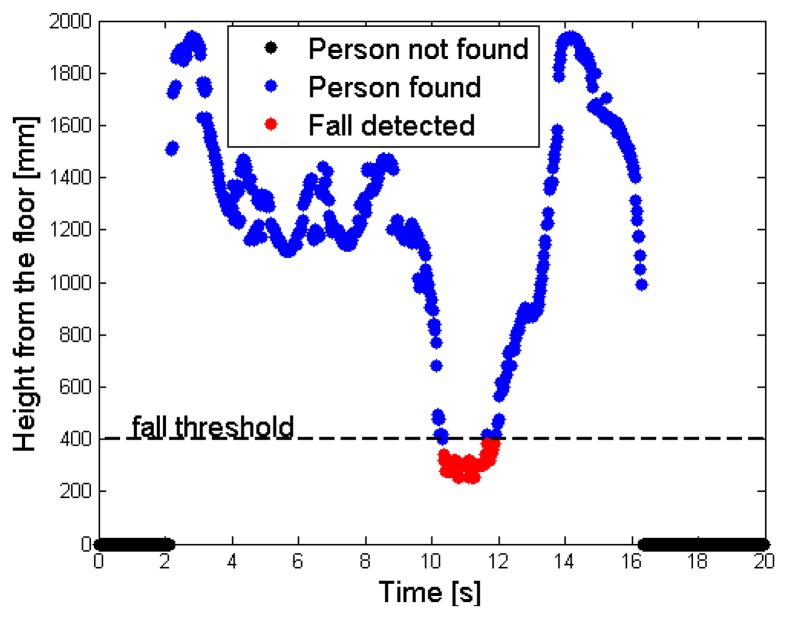
Time sequence of the depth values assumed by the person's central point (*cp_Per_* parameter), in the complex scenario, with the *RF* frame: the fall event is detected.

**Figure 14.**
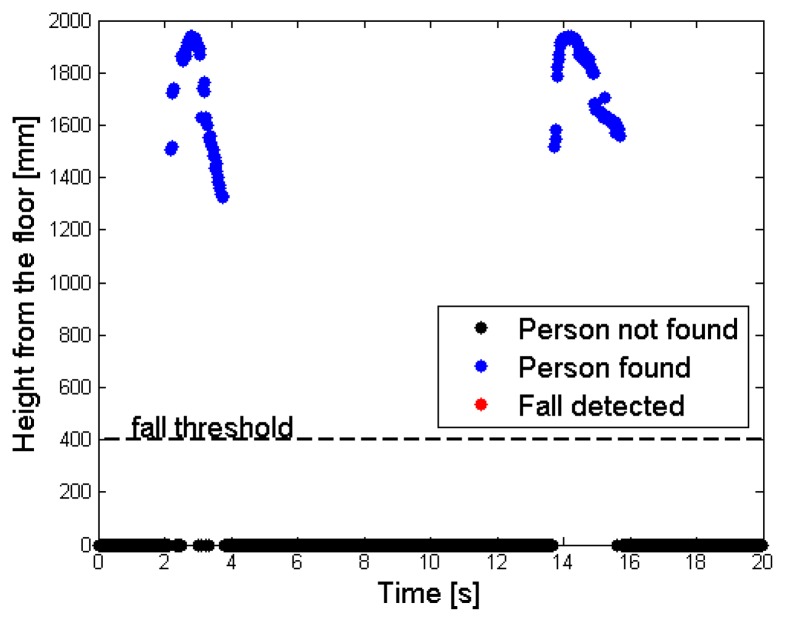
Time sequence of the depth values assumed by the person's central point (*cp_Per_* parameter), in the complex scenario, without the *RF* frame: the fall event is not detected.

Figure 12, on the contrary, displays the results obtained in the same conditions of complex fall, when the *RF* is exploited by the algorithm. In this case, the depth values associated to the person are correctly enhanced, and the distinction between the objects and the person is well evident in the *FF* frame of Figure 12b. Figure 13 has the same meaning of Figure 10: the phase during which the subject enters the area detected by the sensor can be easily noticed, followed by some interactions with the objects, then a fall event. In the final part of the monitored interval, the subject gets up and leaves the scene. The same considerations cannot be applied to Figure 14, obtained when the system tries to track the person without exploiting the *RF* frame. At first the subject is identified, similarly to Figure 13, but then the corresponding super-pixels are merged with the desk blob, forming a unique object. During the fall event, the system finds a new object undergoing the threshold of 400 mm near to the floor, but it cannot understand that it corresponds to a person. The person is detected once again only when he gets up, after the fall event, that has not been notified by any alarm.

## 5. Conclusions

In this work, a method for automatic fall detection using the Kinect^®^ depth sensor in top-view configuration has been proposed. This approach allows detecting a fall event without relying on wearable sensors, and by exploiting privacy-preserving depth data only. Starting from suitably preprocessed depth information, the system is able to recognize and separate the still objects from the human subjects within the scene, through an *ad-hoc* discrimination algorithm. Several human subjects may be monitored through a solution that allows simultaneous tracking. The system performance, verified through experimental tests, confirms:

- the capability of recognizing the human silhouette, during a fall event, even when the subject interacts with objects;
- the potentiality of the algorithm proposed for handling the blob fusions in the depth domain.

A C++ implementation of the proposed system has been realized and tested, in real-time execution, on a desktop PC featuring Windows 7 O.S., Intel i5 processor, and 4 GB RAM. The proposed algorithm is adaptable to several depth sensors, once a 320 × 240 resolution and a 30 fps frame rate are set, thanks to the fact that it only needs raw depth information as input data. Finally, an embedded real time implementation has been also realized on a development platform, featuring Ubuntu Linaro 12.11 O.S., ARM Cortex-A9 Quad Core architecture processor, and 2 GB RAM. In this case, the frame rate supported by the board is slightly lower than 30 fps, but the system still works properly.

With the aim of extending the coverage area that can be monitored, future research activities will focus on improving and optimizing the algorithm performance, to simultaneously handle and coordinate several depth sensors, in an array-like configuration. In particular, the system will be designed to support the tracking of human subjects when crossing areas covered by adjacent sensors in top-view position. Another aspect, currently under investigation, deals with the feasibility of transmitting raw information, or a compressed version of it, captured by several depth sensors, over a possibly wireless data network, to implement a centralized processing of multiple depth information flows, for a distributed monitoring and tracking of human subjects in extended areas.

## Figures and Tables

**Table 1.** Objects within *Mat_Obj* are ordered according to their occurrence through the rows of the *FF* frame.

OI	***x***	***y***	***X***	***y***	***x***	***y***	***x***	***y***	***x***	***y***
**1**	2	4	2	5	0	0	0	0	0	0
**2**	8	9	9	9	7	10	8	10	9	10
**3**	27	11	28	11	29	11	26	12	27	12
**4**	7	16	8	16	9	16	10	16	0	0
**5**	14	18	15	18	16	18	14	19	15	19
**6**	20	25	20	26	21	26	22	26	23	26
**7**	4	31	5	31	6	31	4	32	5	32

**Table 2.** *TrackingInfo* data structure referred to the *k-th FF* frame of Figure 7.

**Blob Index**	**Pers**	**Obj**	**N_pers**	**N_obj**
1	/	1	/	1
2	/	2	/	1
3	1–2	/	2	0
4	/	3	/	1
